# Analyses of hospitalization in Alzheimer's disease and Parkinson's disease in a tertiary hospital

**DOI:** 10.3389/fpubh.2023.1159110

**Published:** 2023-05-04

**Authors:** Sihui Chen, Jiajia Fu, Xiaohui Lai, Yan Huang, Ting Bao, Xueping Chen, Huifang Shang

**Affiliations:** ^1^Department of Neurology, Laboratory of Neurodegenerative Disorders, National Clinical Research Center for Geriatrics, West China Hospital, Sichuan University, Chengdu, Sichuan, China; ^2^Management Center, West China Hospital, Sichuan University, Chengdu, Sichuan, China

**Keywords:** Alzheimer's disease, Parkinson's disease, hospitalization, a tertiary hospital, analyses

## Abstract

**Background:**

To characterize the pattern of hospitalization in patients with Alzheimer's disease (AD) or Parkinson's disease (PD), and compare the differences to see whether AD patients and PD patients have a different picture of hospitalization.

**Methods:**

The clinical features of all consecutive patients from January 2017 to December 2020 were reviewed. We identified AD patients and PD patients from an electronic database in a tertiary medical center.

**Results:**

The study group comprised 995 AD patients and 2,298 PD patients who were admitted to the hospital for the first time, and re-hospitalized 231 AD patients and 371 PD patients were also included. AD patients were older than PD patients when they were hospitalized (*p* < 0.001). AD patients had longer lengths of stay, higher re-hospitalization rates, and higher intrahospital mortality rates than PD patients during hospitalization even after adjusting age and gender. PD patients had higher levels of total cost than AD patients due to the cost of the deep brain stimulation (DBS) insertion. Hospitalizations for AD patients occurred most often in the department of geriatrics, while most PD patients were admitted to the department of neurology. Hospitalization due to the presence of comorbid conditions was much higher in AD patients, but a larger proportion of PD patients were hospitalized due to PD disease itself.

**Conclusions:**

The present study found that AD patients and PD patients have a significantly different picture of hospitalization. It is important to implement different management for hospitalized AD and PD, and different emphasis should be given when establishing primary prevention strategies, informing care needs, and guiding healthcare resource planning.

## Introduction

Alzheimer's disease (AD) is the most common age-related neurodegenerative disease, and Parkinson's disease (PD) is the second most common neurodegenerative disorder. Over the past decades, AD and PD have become a heavy public health burden in China. From 1990 to 2016 in China, the age-adjusted prevalence of dementia reached 5.6%, while the global prevalence increased by 1·7% (1). In 2020 China, it was estimated that 9.83 million had AD, out of the 249.49 million people aged ≥60 years old (2). From 1990 to 2016 in China, the age-adjusted prevalence rates of PD more than doubled, which is the largest increase worldwide (3). It is estimated that the number of PD patients in China will rise to 4.94 million in 2030, accounting for 57% of the total number (4). The hospitalization of AD patients and PD patients influences healthcare utilization and life expectancy. Besides disease-related conditions, respiratory diseases, cardio-cerebrovascular disorders, infection, falls, and bone fractures complaints are the common causes of hospital admission. Despite the importance of hospitalizations in AD and PD, there are as yet no studies to address these issues and to allow direct comparison. Here, we describe the features of the hospital admissions of a geographically defined population of AD and PD patients over 4 years (January 2017-December 2020). The aims of the present study are: (1). to show the characteristics of hospitalization in AD and PD patients in Southwest China; (2). to detect the impact of comorbid conditions in AD and PD patients; (3). to compare the differences in hospitalization between AD patients and PD patients; (4). to identify possible preventive strategies targeting differently to AD and PD.

## Methods

**Figure 1:**
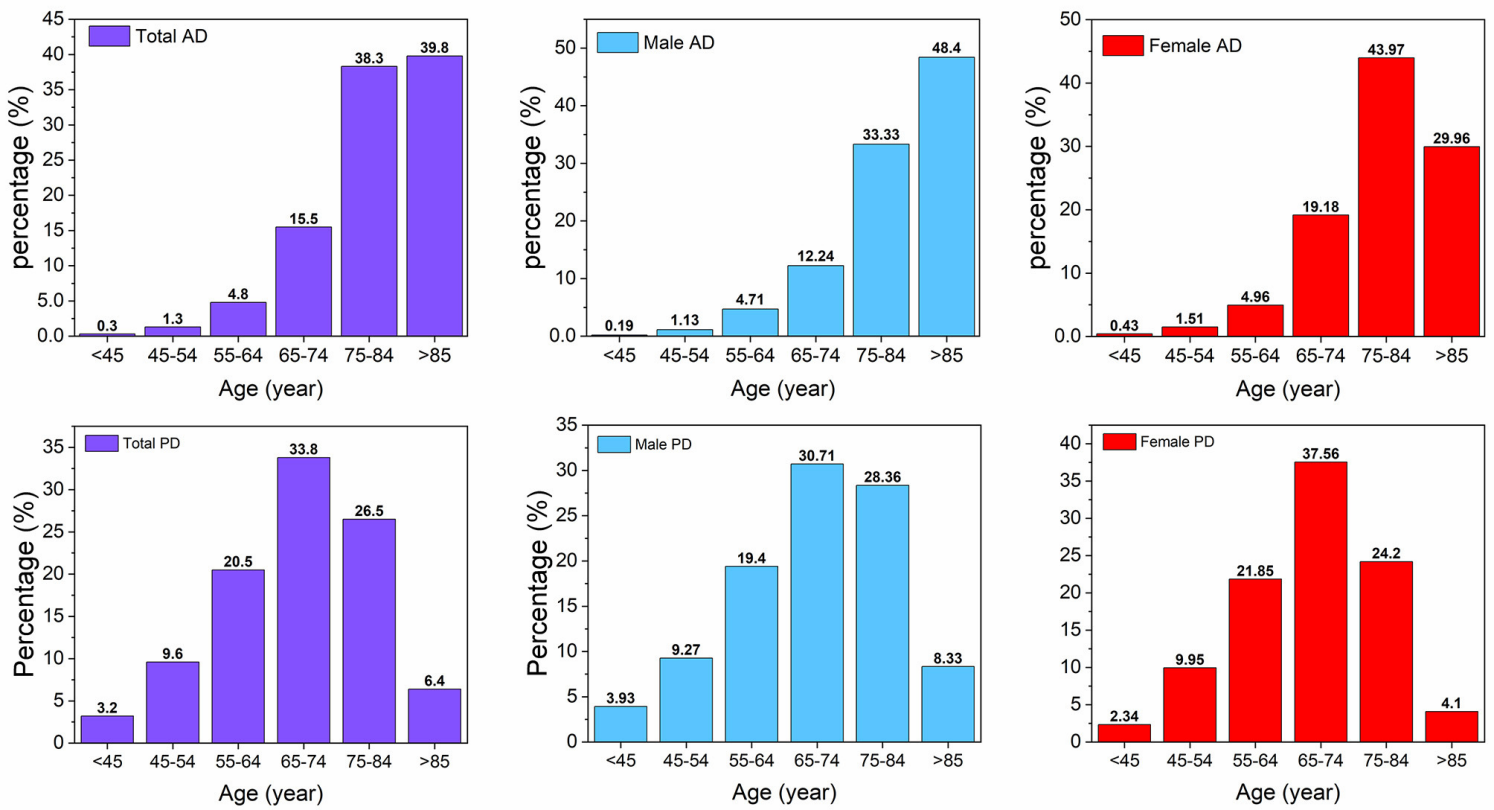
Screening process of AD patients and PD patients. AD, Alzheimer's disease; PD, Parkinson's disease.

West China Hospital (WCH) is a tertiary medical center located in Chengdu city, Sichuan Province, China, with about 300,000 patients discharged from inpatient departments annually. WCH has 4,300 beds and covers an area of more than 470,000 m^2^. All clinical records of patients with the diagnosis of AD or PD discharged from January 2017 to December 2020 were consecutively reviewed by two clinicians trained in neurodegenerative disorders. Medical records of patients with a discharge diagnosis of AD or PD were collected from the computer patient administration system. Other types of dementia, including vascular dementia, frontotemporal dementia, Lewy body dementia, and other extrapyramidal disorders, including Parkinsonism-plus, and vascular or drug-induced Parkinsonism were excluded. We excluded patients diagnosed with both PD and AD (or dementia) at the time of initial screening. Admissions for dialysis and inpatient rehabilitation were also excluded from this analysis. The screening flow was shown in Figure 1. Ethics approval of data collection protocol received from the Ethical Committee of West China Hospital of Sichuan University.

Demographic data, including age at first hospitalization, gender, reasons for hospital admission, admission ward, mean days of hospital stay, surgical procedures and outcomes, were collected in all the AD patients and PD patients. The cause of hospitalization was informed as the main diagnosis, defined as what caused the hospitalization. The main diagnosis was categorized as AD or PD, neuropsychiatric disorders, immune-related disorders, cerebrovascular disease, other neurological diseases (dizziness, headache, encephalitis, hydrocephalus, etc.,), pulmonary infection, urinary infection, sepsis, other infectious diseases (skin infection, digestive tract infection, etc.,), respiratory disease, vascular disorder, gastrointestinal disorders, urological disorders, endocrine and metabolic diseases, tumors, others medical diseases, head trauma, fracture, others trauma. The common reasons for hospital admission were recorded, and the differences in the cause of hospitalization were analyzed between AD and PD patients. Mean days hospitalized were calculated using the following formula: the total in-hospital days of all hospitalizations/the number of hospital admissions during the 4 years. Hospitalization duration was calculated based on the length of hospitalization from admission to discharge. The total costs during the hospitalization were recorded, composed of care services, medical expenses, examination/laboratory test charges, and surgery costs. Reasons for in-hospital deaths were obtained by reviewing medical records.

Between-group differences in proportions for categorical variables were assessed using Wilcoxon rank-sum, Chi-square, or Fisher's exact tests, and Student's t-test was used to compare continuous variables. An analysis of covariance (ANCOVA) was used to compare differences between AD and PD patients in total costs, care services, medical expenses, examination/laboratory test charges, surgery costs, and mean hospital days, with age and sex as covariates. Bonferroni correction was applied to optimize for multiple tests.

Logistic regression analyses were performed to assess associations of in-hospital deaths or re-hospitalizations with the main diagnosis while adjusting for potential confounding variables. These analyses resulted in odds ratios (OR) with 95% confidence intervals (CI). Two-sided *p*-values < 0.05 were considered statistically significant.

## Results

### Admission distribution

**Figure 2:**
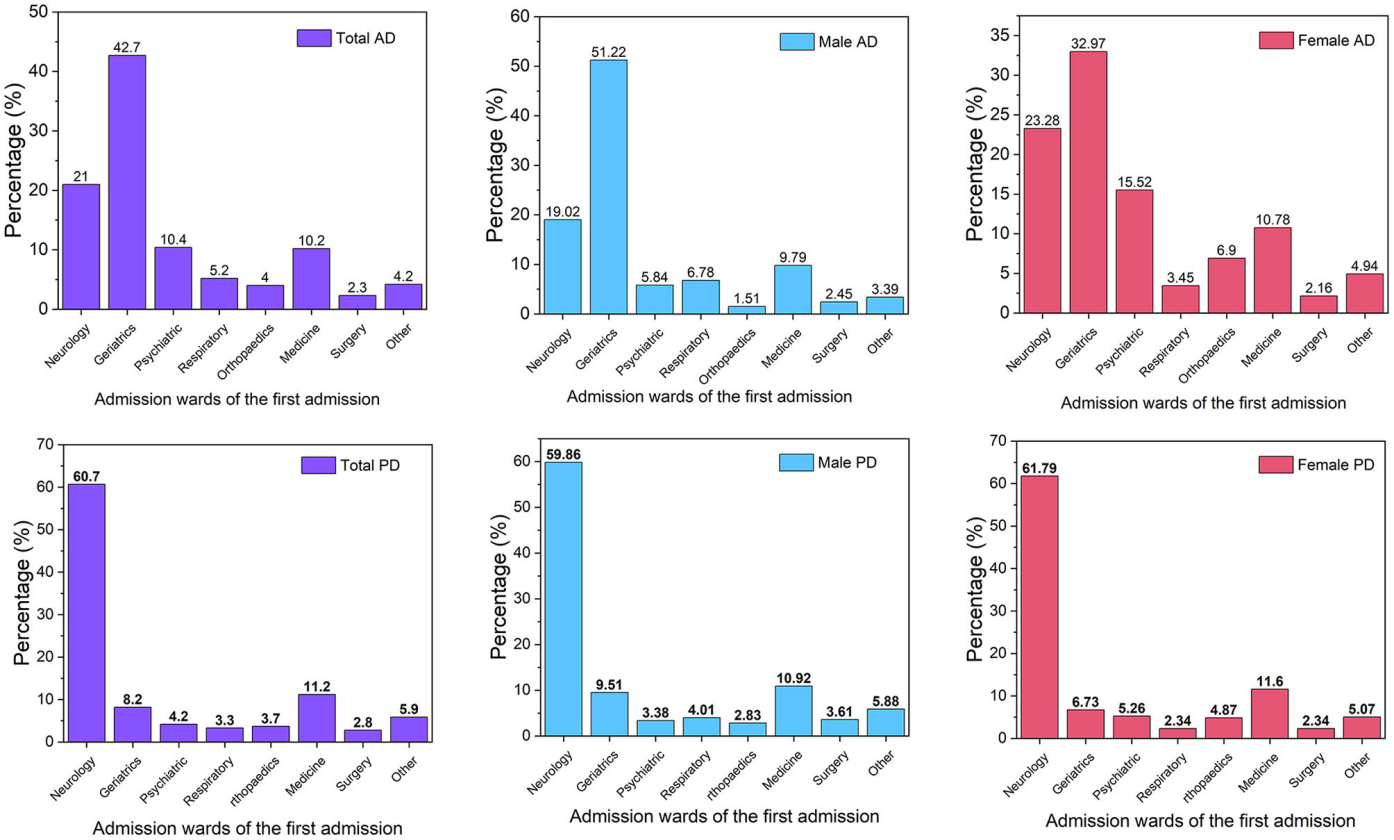
Age distribution of AD and PD on first admission. AD, Alzheimer's disease; PD, Parkinson's disease.

During the study period, there were 3,293 first admissions involving 995 AD patients and 2,298 PD patients (Figure 2). The mean age at first hospitalization was 80.78 ± 0.30 years in AD patients, and 68.36 ± 0.24 years in PD patients; AD patients were significantly older than PD patients when first admitted to the hospital (*p* < 0.001). The female/male ratio was 464/531 in AD patients and 1,025/1,273 in PD patients, and there were no significant differences in gender ratio between AD and PD patients (χ^2^ = 1.154, *P* = 0.283). We only found that the gender ratio was significantly different in those patients aged between 75 to 84 years (χ^2^ = 15.53, *p* < 0.001), and this finding was shown in Figure 2 (Supplementary Table S1). In AD patients, 79.1% were aged ≥75 years old when they were first hospitalized, while in PD patients, 33.8% were aged between 65 to 74 years old, and 26.5% were aged between 75 to 84 years old. When sex and age were added as covariates, the results of ANCOVA analysis showed that the difference in mean hospitalization duration of the first admission between AD patients and PD patients was still significant (19.18 ± 0.80 vs.11.01 ± 0.27, *F* = 71.71, *p* < 0.01).

### Admission wards

**Figure 3:**
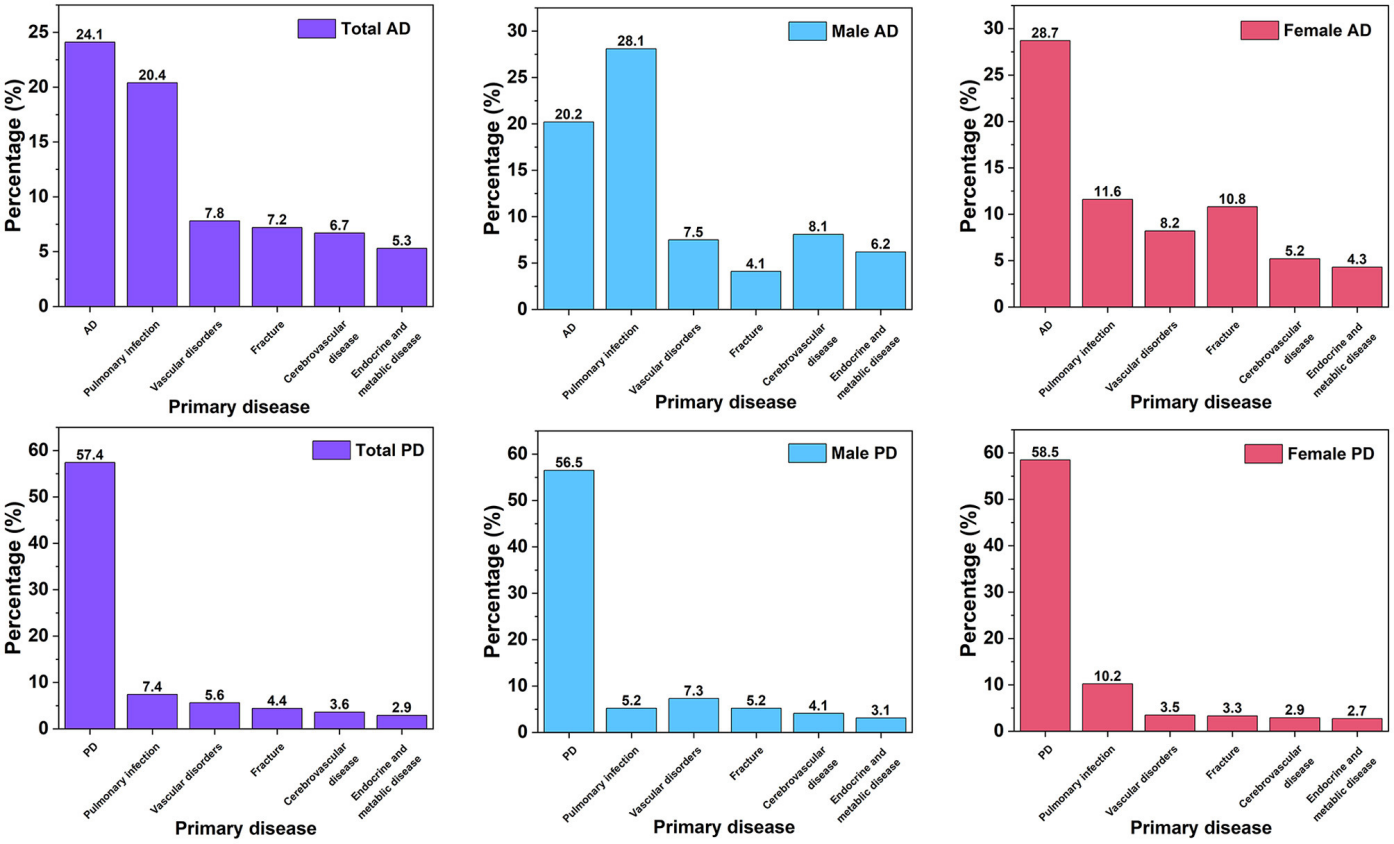
First admission department distribution of AD and PD. AD, Alzheimer's disease; PD, Parkinson's disease.

Admission wards of the first admission for AD patients and PD patients were listed in Figure 3. In AD patients, 42.7% were admitted to the department of geriatrics, while in PD patients, 60.7% were admitted to the department of neurology (Supplementary Table S2). Both AD and PD patients with different gender had similar distributions in the admission ward (Supplementary Table S1).

### Admission reason

**Figure 4:**
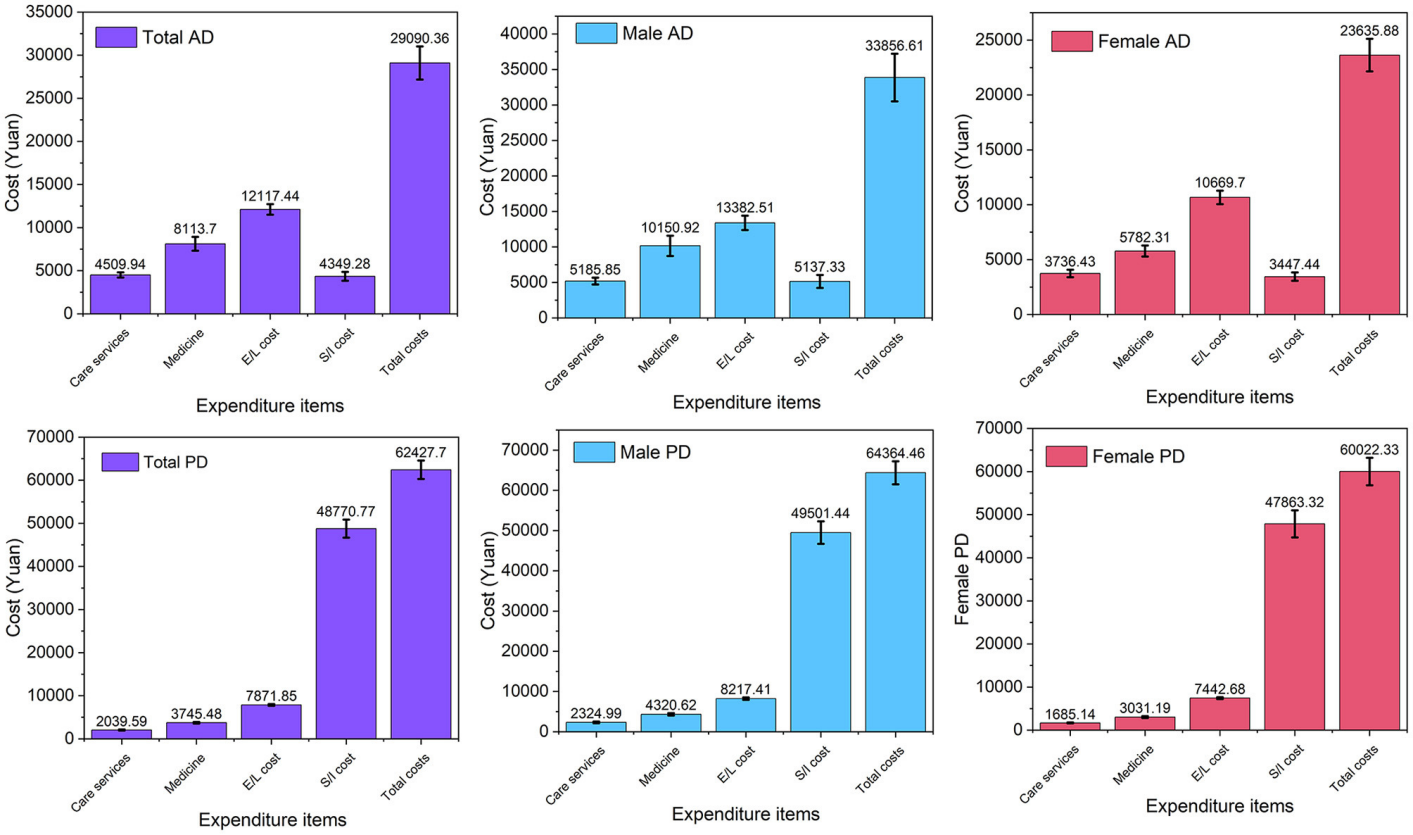
The main diagnostic distribution of AD and PD on first admission. AD, Alzheimer's disease; PD, Parkinson's disease; OND, Other neurological diseases; OID, Other infectious diseases; OMS, Other medical disorders; OT, Other trauma.

The primary diagnosis for the first admission in AD patients and PD patients was listed in Supplementary Table S3, and the top six main reasons for hospital admission were shown in Figure 4. In AD patients, the presence of comorbid conditions caused 755 out of 995 admissions (75.9%), while the remaining AD patients were admitted due to the main diagnosis of AD. The most common cause of the first hospitalization in AD patients was AD disease itself (24.1%); the following main causes were pulmonary infection (20.4%), vascular disorder (7.8%), and fracture (7.2%). In female AD patients, 28.7% were caused by AD disease itself, followed by pulmonary infection (11.6%), fracture (10.8%), and vascular disorder (8.2%). In male AD patients, 28.1% had the main diagnosis of pulmonary infection, followed by AD itself (20.2%), cerebrovascular disease (8.1%), and vascular disorder (7.5%). In PD patients, 979 out of 2,298 admissions (42.6%) were caused by the presence of comorbid conditions, while the remaining PD patients were admitted for a diagnosis of PD or a complication directly related to PD (motor fluctuation or dyskinesia). The most common cause of the first hospitalization was PD disease itself (57.4%); the following main causes were fracture (7.4%), pulmonary infection (5.6%), and neuropsychiatric disorders (4.7%). Of male PD patients, 56.5% were caused by PD disease itself (56.5%), followed by pulmonary infection (7.3%), fracture (5.2%), and vascular disorder (5.2%). In female PD patients, 58.5% had the main diagnosis of PD, followed by fracture (10.2%), neuropsychiatric disorders (6.1%), and pulmonary infection (3.5%). Hospitalization due to the presence of comorbid conditions was much higher in AD patients compared with PD patients (χ^2^ = 308.43, *p* < 0.001). A larger proportion of PD patients were hospitalized due to PD disease itself, while AD patients had a higher proportion of pulmonary infection and vascular disease than PD patients while hospitalized (Supplementary Table S3).

### Hospitalization costs

**Figure 5:**
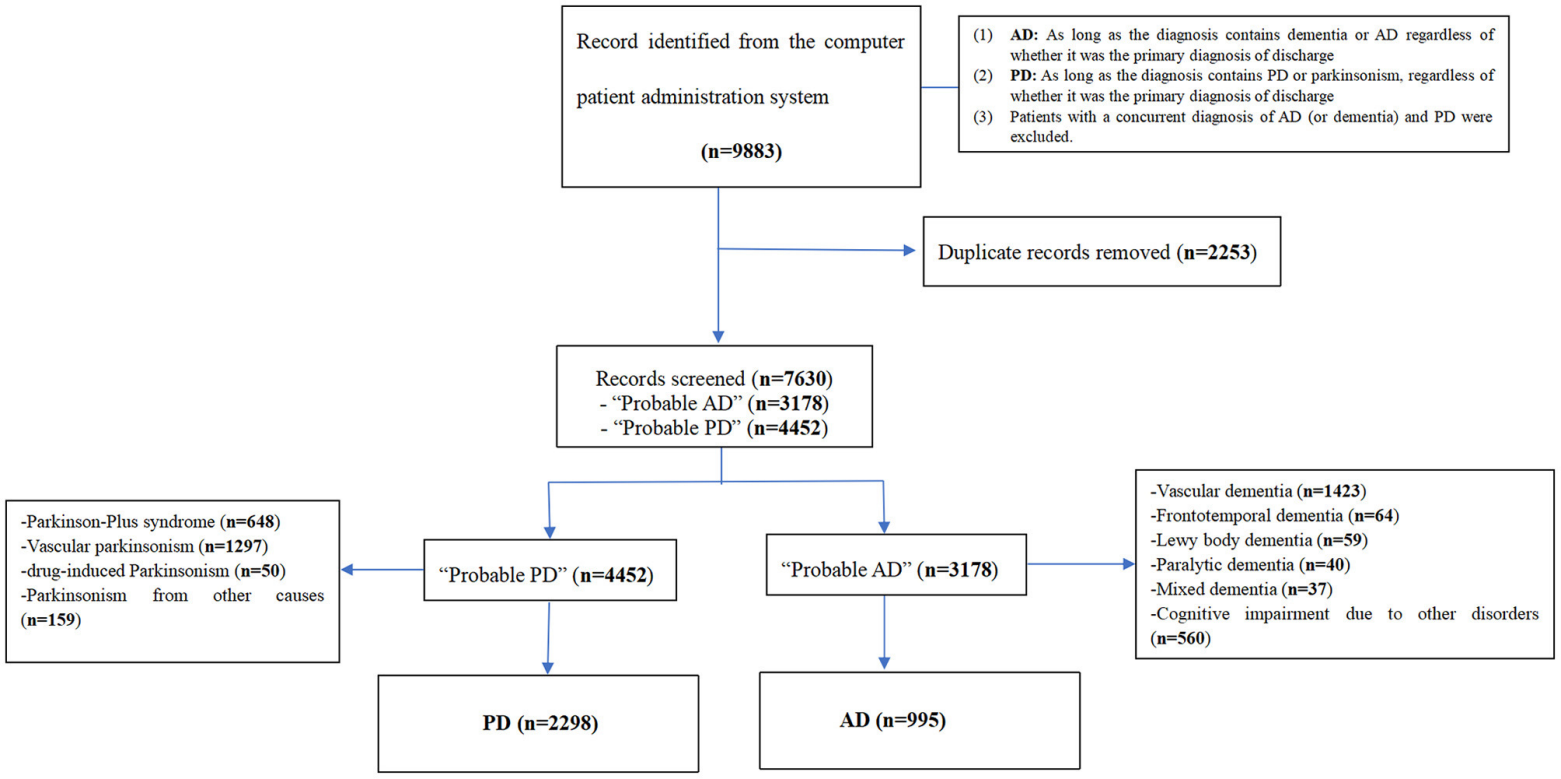
Comparison of total first-admission costs between AD and PD. AD, Alzheimer's disease; PD, Parkinson's disease; E/I cost, examination/laboratory tests; S/I cost, surgery/inventory cost.

The total costs from the first admission were 29,090.36 ± 1,923.21 and 62,427.70 ± 2,125.80 Yuan in AD patients and PD patients, respectively. The total cost of the first admission was significantly higher in PD patients than that in AD patients, even after adjusting for age and sex (*F* = 18.11, *p* < 0.001) (Figure 5 and Supplementary Table S4). ANCOVA analysis also showed that AD patients had higher expenses in care services (*F* = 24.63, *p* < 0.001), medicine (*F* = 15.88, *p* < 0.001), and examination/laboratory tests (*F* = 335.24, *p* < 0.001) than PD patients. In addition, 422 out of 2,298 PD patients (18.40%) were admitted for DBS insertion. Therefore, the surgery cost was significantly higher in PD patients than in AD patients (*F* = 16.81, *p* < 0.001). When PD patients who received DBS insertion were removed, there was no significant difference in the cost of surgery service for AD patients and PD patients (4,349.28 ± 523.13 vs. 4,628.65 ± 316.06, *F* = 0.40, *p* = 0.526).

### Outcomes

#### Re-hospitalization

During the study period, 231 out of 995 AD patients (23.2%) and 371 out of 2,298 PD patients (16.1%) were re-hospitalized, and AD patients had a statistically higher re-hospitalization rate than PD patients (χ^2^ = 23.24, *p* < 0.001). This difference in re-hospitalization was also significant in male patients (χ^2^ = 141.77, *p* < 0.001), but was not significant in female patients (χ^2^ = 3.47, p = 0.063). The mean re-hospitalization duration was 25.06 ± 1.72 days in AD patients and 11.97 ± 0.48 days in PD patients, and AD patients had a significantly longer duration of re-hospitalization than PD patients even after adjustment for age and sex (*F* = 33.07, *p* < 0.001).

The distribution of re-hospitalization for AD patients and PD patients by age and sex was listed in Supplementary Table S5. The age at re-hospitalized for AD patients was significantly older than that for PD patients (86.06 ± 7.30 vs. 72.05 ± 11.87, *p* < 0.001). Among AD patients, 67.5% were aged ≥85 years, but among PD patients, only 14.6% were older than 85 years (*p* < 0.01). The most common re-hospitalized department for AD patients was the department of geriatric (74.9%), while in PD patients the most common re-admitted department was the department of neurology (38.0%). Admission wards of re-hospitalization for AD patients and PD patients were all listed in Supplementary Table S6. Comorbidities were present in 85.3% (179/231) of AD patients, and 63.9% (237/371) of PD patients when re-admitted, and a significantly higher comorbidity rate was found in AD patients (χ^2^ = 12.347, *p* < 0.001). The most common cause of re-hospitalization was pulmonary infection (32.0%), followed by AD itself (14.7%), vascular disorder (8.7%), and fracture (8.2%). In re-hospitalized PD patients, the most common cause was the PD disease itself (36.1%), followed by fracture (12.1%), pulmonary infection (11.9%), and tumors (7.8%). The main diagnosis for re-hospitalization in AD patients and PD patients was listed in Supplementary Table S7. The total re-hospitalization costs were 30,911.96 ± 2,453.11 and 43,112.32 ± 4,196.12 Yuan in AD and PD patients, respectively. The total cost of re-hospitalization was significantly higher in PD patients than that in AD patients (*p* < 0.001), but this difference was not statistically significant after we adjusted sex and age (*F* = 0.777, *p* = 0.379). There were 37 out of 371 PD patients (9.97%) re-admitted for DBS insertion. Therefore, the surgery costs were significantly higher in PD patients than in AD patients (*p* < 0.05), and this difference was more pronounced in male patients (*p* < 0.01). Interestingly, there was no significant difference between first admission and re-hospitalization costs in AD patients (*p* > 0.05). However, in PD patients, the cost for the first admission was significantly higher than the cost of re-hospitalization (*p* < 0.01), and more PD patients received DBS surgery at first hospitalization compared to re-admission (18.40% vs. 9.97%, χ^2^ = 15.79, *p* < 0.001). The hospitalization costs for re-hospitalization in AD patients and PD patients were listed in Supplementary Table S8. The mean length of hospital stay for the re-admissions in AD patients was significantly longer than that in PD patients (18.79 ± 0.70 vs. 10.87 ± 0.24, *p* < 0.001), and the difference remained statistically significant after adjusting for age and sex in ANCOVA analysis (*F* = 25.76, p < 0.001).

#### Intrahospital mortality

Concerning intrahospital mortality, 48 out of 995 (4.82%) AD patients died, and 42 out of 2,298 (1.80%) PD patients were deceased. The mortality rate was significantly higher in AD patients than that in PD patients (χ^2^ = 23.452, *P* < 0.001). The top three causes of death in AD patients included AD itself (17/48, 35.42%), pulmonary infection (10/48, 20.83%), and fracture (9/48, 18.75%). In PD patients, the top three causes of death were pulmonary infection (21/42, 50%), gastrointestinal disorders (5/42, 11.90%), and PD itself (4/42, 9.50%). There was no significant difference in the proportion of the most common in-hospital causes of death between the two groups (χ^2^ = 1.953, *p* = 0.162). The gender-specific analyses found that the most common cause of death in male AD patients was pulmonary infection (9/25, 36.00%) and AD itself (9/25, 36.00%), but in female AD patients, it was AD itself (8/23, 34.78%) and fracture (8/23, 34.78%). However, pulmonary infection rather than PD itself was the most common cause of death in both male and female PD patients (17/29, 58.60% vs. 4/13, 30.80%).

Logistic regression models were developed to evaluate the variables affecting re-hospitalization (Supplementary Table S9) and intrahospital mortality in AD patients and PD patients (Supplementary Table S10). The result showed that re-hospitalization of AD and PD patients was associated with age, hospitalization duration, neurological hospitalization, and surgical procedures in the first admission. The intrahospital death of PD patients was found to be correlated to age, comorbidity in the hospital, neurological hospitalization, and repeated hospitalization.

## Discussion

Several studies have demonstrated the pattern of hospitalization in AD or PD patients. However, knowledge about hospitalized AD and PD patients and comparing the differences has been limited. The present study described the hospitalization of patients with AD and PD discharged from January 2017 to December 2020 in a tertiary medical center and found that AD patients and PD patients have a significantly different picture of hospitalization.

### Admission distribution

We found the mean age at first admission was 80.78 years in AD patients, which was older than that in PD patients (68.36 years). A retrospective study from the United Kingdom found that the mean age at primary hospitalization was 84.5 years in dementia patients (5). AD is predominantly a disease of older adults, and most of the reasons for hospital admission might have been related to age, AD and its associated comorbidities. Therefore, it is important to focus attention on the age at admission that is specifically associated with hospital admission in AD patients. One reason is that the true level of awareness and knowledge about AD in China is likely to be far lower. In China, dementia continues to be seen by many as an inevitable and natural part of the aging process. Therefore, knowledge about AD can bring about earlier assessment and diagnosis, particularly around addressing modifiable risk factors and the potential for dementia prevention. In PD patients, we noticed that 3.2% were aged ≤ 45 years old when they were first hospitalized, and 9.6% were aged between 45 to 54 years old. Studies have shown that young-onset PD (YOPD in adults under 40 years of age) has been estimated to account for up to 10% of PD patients (6, 7). YOPD is an important subgroup of PD patients, and PD patients are significantly younger when admitted to the hospital.

One study found that the mean duration of stay was 9.7 for PD patients and 9.2 days for controls; the difference was not significant (8). A previous study looked at admissions of PD patients to a general hospital and found that the average length of stay was 11.7 days in PD patients, compared to 8.7 for other adults (9). A prospective cohort study found that the mean length of stay per admission was 10 days in PD patients and 10.5 days in controls, and it was also found that the mean duration of stay in PD patients was seven days, which was longer than that in control patients (10). A systematic review found that PD patients were generally 2–14 days longer hospitalized than non-PD patients (11). For dementia patients, one study found that the mean duration of the hospital stay was longer in patients with dementia (13.4 vs. 10.7 days) (12). In our study, the mean length of stay was longer in AD patients than that in PD patients (18.79 vs. 10.87 days), and this finding may be related to the higher presence of comorbid conditions in AD patients.

### Admission wards

A 4-year hospital discharge database record in Spain showed that internal medicine departments discharged 44% of identified patients with dementia, neurology departments 22%, and psychiatry departments 21% (12). In the present study, we found that 42.7% of AD patients were admitted to the department of geriatrics, followed by neurology departments (21%) and psychiatry departments (10.4%). We also found that most AD patients were not admitted to the hospital for dementia but for other reasons, and that the impact of comorbidity was higher in AD patients than in PD patients. AD patients were hospitalized primarily for medical reasons, including pulmonary infection, vascular disorder, and fracture. Therefore, they are more often admitted to the geriatrics department because the target population of the geriatrics department is older adults with multi-comorbid conditions. When AD patients are admitted to the hospital, their conditions are not limited exclusively to the AD disease itself, and their diseases were progressed to a certain stage. Hospital admissions of AD patients are often problematic, especially when patients are admitted to non-neurological wards. As most non-neurologically educated healthcare personnel are not professionals in AD diagnosis and treatment, protocols would be helpful to improve the care of AD patients in such environments. Guidelines to guide physicians in the hospital environment could be helpful. Hospitals should implement specific programs to identify AD patients earlier and integrated treatment to the comorbidities. In addition, specific care plans should be designed to manage those AD patients who are admitted to the department of geriatrics, which is not specialized in managing AD patients. However, PD patients have less comorbidity than AD patients when they are hospitalized. A systematic review analyzed the factors that lead to hospitalization in PD patients and found that PD-related problems were the most common cause of admission to a neurological ward due to worsening motor or non-motor symptoms (13). This finding contributes to our understanding of the different approaches to the management of AD patients and PD patients in a hospital setting.

### Admission reason

The included AD patients had more hospitalizations caused by comorbid conditions than PD patients. Previous studies have shown that comorbidity and higher age were associated with an increased risk of hospitalization in patients with dementia (14, 15). The incidence rates of falls, injury, and infection were higher in subjects with AD than in those without AD (16). A recent meta-analysis found that there was strong evidence that admission of people with dementia was strongly associated with older age, and was moderately associated with multimorbidity, but dementia severity alone was not associated with admission (17). A retrospective 6-year study identified the most common cause of admission in dementia patients was severe respiratory disorder (respiratory failure and complicated infections) (12). Similarly, a retrospective study found that acute lower respiratory tract infection, pneumonia, and fractured neck of femur diagnoses were more prevalent in AD patients compared to controls when they were admitted (5). A cohort study also found that the most common hospitalization discharge diagnoses among patients with dementia were urinary system disorders, pneumonia, and fracture of the femur (18). An earlier systematic review of reasons for admissions for people with dementia also found that people with dementia were more likely to have been admitted to hospitals for respiratory and urological infections, falls, and fractures than inpatients without dementia (19). The high proportion of pulmonary infections in hospital admission for AD patients may be due to a lack of physical exercise, mobility, and self-neglect, and perhaps also impaired swallowing function in these patients, which increases the risk of aspiration pneumonia (20). An Italian hospital-based study obtained data from 51,838 consecutive computerized discharge records and found a higher prevalence of cerebrovascular disease, pneumonia, and hip fracture among the primary diagnoses in dementia patients (21). Dementia patients also had more acute cardiac events than non-dementia cases (22). These findings suggested a greater load of comorbidity in AD patients. Therefore, early recognition and management of respiratory infections and meticulous fall prevention are valuable future intervention strategies in AD patients. Obviously, a large proportion of hospital admissions caused by muti-comorbidity of advanced AD may be avoidable by early intervention.

The present study found that the proportion of patients admitted for PD disease as their primary diagnosis was larger than AD patients. A prospective cohort study also found that the most common cause of admissions to the hospital was PD disease itself, followed by vascular disease, trauma/fracture, and pulmonary infection, and a retrospective study analyzed the precipitants for admission to a large teaching hospital in PD patients and found that the most common primary diagnosis was PD disease itself, followed by falls and fractures, pneumonia, and gastrointestinal complications (23, 24). PD and its related problems appear responsible for about 25% of acute hospitalizations in PD patients (13). Exacerbation of PD-related symptoms can be avoided by frequent review of PD patients and appropriate adjustment of their medications to improve motor and non-motor function and manage drug-related side effects. Furthermore, poor motor control is associated with hospital admission, and optimizing PD symptom control through medication compliance may help reduce hospital admissions. A systematic review aiming to identify interventions to reduce hospitalization in PD found that frequent neurologist consultation and compliance with PD medication may reduce hospital admissions (25). When PD patients are admitted to the hospital due to PD disease itself, professional neurological consultations and treatments should be provided for medication adjustments, earlier detection, and management of complications. Therefore, in PD patients, frequent outpatient reviews with an emphasis on medication adjustment to maintain good mobility may help reduce hospitalization.

The present study found that besides PD disease, fracture, pulmonary infection, and neuropsychiatric disorders were common reasons for hospitalization. A systematic review confirmed that PD-related motor complications, psychiatric problems, infections, and falls were the top four main reasons for admission to a neurological ward (13). Another systematic review also summarized the causes for admission, and the leading causes included injuries (many with fractures), infections (mainly pneumonia and urinary tract infections), and poor control of PD (11). Compared to controls, admission for aspiration pneumonia, trauma/fracture, and psychosis were found to be higher in PD patients (26). Similarly, a previous study identified falls, fractures, infections, and cognitive and motor decline as risk factors for unplanned hospital admissions in patients with PD (27). Normally, falls and fractures can occur in PD due to postural instability, poor motor symptom control, and drug-related side effects. Therefore, early recognition and management of respiratory infections and meticulous fall prevention are valuable future intervention strategies in PD patients. Reducing the number of admissions might be achieved by extra attention to fall prevention, adequate drug regulation with acuity for side effects, and preventing and recognizing early symptoms of infections.

### Hospitalization costs

A previous study found that the average daily cost per PD patient in 2005 was NOK 4500 (EUR 548) (28). The present study's average daily cost for each PD patient was RMB 5,675 YUAN (EUR 832). However, it should be noted that PD patients included in the present study represented a select group of people with PD attending specialist centers of care, as reflected by the rate of DBS (18.40%). Thus, this cohort may have had more advanced diseases and more complex medical issues. Therefore, the surgery costs of PD patients were higher in PD than in AD due to the higher cost of DBS devices. However, AD patients had higher expenses in care services, medicine, and examination/laboratory tests than PD patients, and the differences in these hospitalization costs could be explained by the longer length of stay and higher presence of comorbidities of patients with AD compared to those with PD.

### Outcomes

A previous study analyzed the hospital-based discharge database records and found that the intrahospital mortality in dementia patients was 19.3%, which was higher than that in patients without dementia (8.7%) (12). Another study also found that the intrahospital mortality rate in dementia patients was higher than that in non-demented patients (10.5 vs. 8.7%) (21). Dementia was associated with a higher rate of admissions to hospitals, and dementia was an independent risk factor for intrahospital death (12, 21). A Meta-analysis of hospital administrative database studies showed that the mortality in dementia patients was 15.3% as compared to 8.7% in non-dementia cases (22). In the present study, we found that intrahospital mortality in AD patients was 4.82%, significantly higher than in PD patients (1.80%). Our samples of patients reflected that AD patients tended to be older, and were diagnosed at a later stage than PD patients. Our AD patients also had higher levels of comorbid conditions and a longer hospital stay, which may have influenced their higher levels of intrahospital death. Proper diagnosis and timely treatment should be available for AD patients (29, 30). However, a previous study evaluated the status of dementia diagnosis and treatment in China and found that a tremendous number of dementia patients were being overlooked (31). According to the “**World Alzheimer Report 2019. Attitudes to dementia”**, 95% of the public believe that they will develop dementia in their lifetime, and 2/3 of the people believe that dementia is a normal aging process. Therefore, there is an urgent need first to raise public awareness of AD. Second, it is important to implement a dementia training program to enhance the capacity of neurologists to manage AD patients (16). Third, setting up memory clinics to screen dementia at general hospitals could be useful to dementia practice in China (16, 32). Fourth, establish a multidisciplinary treatment (MDT) model and a one-stop clinic to develop personalized treatment plans for each AD patient.”

In the present study, the re-hospitalization of PD patients was related to the surgical procedures during the first admission. Similarly, another study also suggested that DBS was a significant predictor of a new encounter with a hospital (26). According to previous research, intrahospital mortality ranged from 4 to 39% in PD patients (9, 10, 33, 34). A previous study reported that intrahospital mortality was 6% in both PD patients and matched controls (8). In our study, mortality was lower than those reported previously in other cohorts, which may be related to the standardized management of PD patients in our center, particularly in neurology hospitalizations. First, our hospital took the lead in establishing the largest PD one-stop treatment center in Southwest China, which integrated multidisciplinary resources, and provided a number of cutting-edge treatments, including transcranial Doppler ultrasound, tremor gait analysis and DBS surgery. Second, we have built a database of PD patients, and conducted regular follow-up visits with each PD patient. During the follow-up visits, we assessed the motor function, as well as cognition, life skills, mental and emotional status, and caregiver burden. Through regular follow-up visits and standard assessments, we enhanced the doctor-patient interaction and promoted individualized treatment of the disease. Third, WCH also launched online clinics and teleconsultations to enable early intervention to reduce the admission for complications. In addition, this difference also depends on the different hospital facilities and the level of medical treatment.

## Strengths and limitations

Considering limitations, despite a large number of the study population, the present analysis is based on electronic records from a single hospital. Thus, it needs to be replicated in different samples. Considering reasons for hospitalization, these data were derived from the discharge diagnoses recorded in the electronic medical records system. It was impossible to distinguish the complications that arose during the hospital stay from the reasons for the initial admission. Concerning comorbid conditions, newly diagnosed persons may have less disease-related comorbidity. The diagnosis of AD or PD can be challenging, and it is impossible to monitor the quality of each diagnosis; however, we know that each diagnosis is made by senior clinicians during hospital admission and reviewed carefully when the patient is discharged. Future studies with a multi-centered approach would allow comparison of a larger group of patients with AD or PD and make our results more generalizable.

## Conclusions

In this study, we identified many clinically important issues that AD patients and PD patients experienced differently. AD patients were older than PD patients when they were hospitalized; AD patients had longer lengths of stay during hospitalization, higher re-hospitalization rate, and higher intrahospital mortality rate than PD patients. PD patients had higher levels of total cost than AD patients during hospitalization due to the cost of DBS insertion. Hospitalizations for AD patients occurred most often in the department of geriatrics, while most PD patients were admitted to the department of neurology. Hospitalization due to the presence of comorbid conditions was much higher in AD patients. AD Patients had a higher proportion of pulmonary infection and vascular disease than PD patients while hospitalized, but a larger proportion of PD patients were hospitalized due to PD disease. In AD patients, raising public awareness and knowledge about dementia, establishing memory clinics, developing a dementia training program, active anti-dementia treatment, and implementing preventative measures for comorbidity are important interventions to reduce the need for hospital admission and mortality. However, the most effective measure to reduce the hospitalization rate of PD patients is to maintain good mobility, manage drug side effects, and control motor complications. The results of the present study may be useful in establishing primary prevention strategies, informing care needs, and guiding future healthcare resource planning and allocation.

## Data availability statement

The original contributions presented in the study are included in the article/Supplementary material, further inquiries can be directed to the corresponding author.

## Ethics statement

The study was approved by the Ethics Committee of West China Hospital of Sichuan University.

## Author contributions

SC, JF, and XL contributed to the compilation of articles and data analysis. YH and TB contributed to the selection and data entry. XC and HS contributed to the review, editing, and scientific research thinking and methods. All authors have read and approved the manuscript.
