# Supplementary material for: Analyses of hospitalization in Alzheimer's disease and Parkinson's disease in a tertiary hospital
Source: Front Public Health. 2023 May 4;11:1159110. doi: 10.3389/fpubh.2023.1159110 (PMC10192859; doi:10.3389/fpubh.2023.1159110)
Supplement: Supplementary file 1 [file Data_Sheet_1.pdf]

Table S1

Age distribution of AD and PD on first admission.

Age (year)	first admission					
	AD			PD		
	Total	Male (%)	Female (%)	Total	Male (%)	Female (%)
< 45	3 (0.3)	1 (0.19)	2 (0.43)	74 (3.2)	50 (3.93)	24 (2.34)
45-54	13 (1.3)	6 (1.13)	7 (1.51)	220 (9.6)	118 (9.27)	102 (9.95)
55-64	48 (4.8)	25 (4.71)	23 (4.96)	471 (20.5)	247 (19.40)	224 (21.85)
65-74	154 (15.5)	65 (12.24)	89 (19.18)	776 (33.8)	391 (30.71)	385 (37.56)
75-84	381 (38.3)	177 (33.33)	204 (43.97)	609 (26.5) *	361 (28.36)	248 (24.20)
85+	396 (39.8)	257 (48.40)	139 (29.96)	148 (6.4)	106 (8.33)	42 (4.10)
All ages	995 (100)	531 (100)	464 (100)	2298 (100)	1273 (100)	1025 (100)

Male (%): the number of male patients as a percentage of the total male population at that age level; Female (%): the number of female patients as a percentage of the total female population at that age level; For those in the same age range, *$p < 0.05$ suggested a statistically significant difference between the gender distribution of AD and PD patients.

Table S2

First admission department distribution of AD and PD.

Admission wards	first admission					
	AD			PD		
	Total	Male (%)	Female (%)	Total	Male (%)	Female (%)
Neurology	209 (21)	101 (19.02)	108 (23.28)	1396 (60.7) *	762 (59.86)	634 (61.79)
Geriatrics	425 (42.7)	272 (51.22)	153 (32.97)	189 (8.2) *	121 (9.51)	69 (6.73)
Psychiatric	103 (10.4)	31 (5.84)	72 (15.52)	97 (4.2) *	43 (3.38)	54 (5.26)
Respiratory	52 (5.2)	36 (6.78)	16 (3.45)	75 (3.3) *	51 (4.01)	24 (2.34)
Orthopedics	40 (4.0)	8 (1.51)	32 (6.9)	86 (3.7)	36 (2.83)	50 (4.87)
Infectious	8 (0.8)	1 (0.19)	7 (1.51)	9 (0.4)	7 (0.55)	2 (0.19)
Rehabilitation	7 (0.7)	2 (0.38)	5 (1.08)	41 (1.8) *	21 (1.65)	20 (1.95)
urology	6 (0.6)	2 (0.38)	4 (0.86)	26 (1.1)	22 (1.73)	4 (0.39)
Medicine	102 (10.2)	52 (9.79)	50 (10.78)	258 (11.2)	139 (10.92)	119 (11.6)
Surgery	23 (2.3)	13 (2.45)	10 (2.16)	64 (2.8)	46 (3.61)	24 (2.34)
Intensive care unit	8 (0.8)	7 (1.32)	1 (0.22)	11 (0.5)	7 (0.55)	4 (0.39)
Other	12 (1.2)	6 (1.13)	6 (1.29)	46 (2.0) *	18 (1.41)	22 (2.14)
Total	995 (100)	531 (100)	464 (100)	2298 (100)	1273 (100)	1026 (100)

Male (%): the number of male patients in this department as a percentage of the number of males in all departments; Female (%): the number of female patients in this department as a percentage of the number of all departments with female patients; For AD and PD patients hospitalized in the same department, *$p < 0.05$ indicated a significant difference in the sex distribution of AD and PD patients.

Table S3

Distribution of primary diagnoses of AD and PD at first admission.

		first admission					
Primary diagnosis	Specification	AD			PD		
		Total	Male (%)	Female (%)	Total	Male (%)	Female (%)
Neurological	AD or PD	240 (24.1)	107 (20.2)	133 (28.7)	1319 (57.4) *	719 (56.5)	600 (58.5)
	Neuropsychiatric disorders	40 (4.0)	14 (2.6)	26 (5.6)	107 (4.7)	44 (3.5)	63 (6.1)
	Neurogenetic/Immune related disorders	46 (4.6)	21 (4.1)	24 (5.2)	31 (1.3) *	16 (1.3)	15 (1.5)
	Cerebrovascular disease	67 (6.7)	43 (8.1)	24 (5.2)	67 (2.9) *	39 (3.1)	28 (2.7)
	Others	14 (1.4)	6 (1.1)	8 (1.7)	34 (1.5)	23 (1.8)	11 (1.1)
	Total	407 (40.90)	191 (36.1)	215 (46.3)	1558 (67.80) *	841 (66.1)	716 (69.9)
Infectious	Pulmonary infection	203 (20.4)	149 (28.1)	54 (11.6)	129 (5.6) *	93 (7.3)	36 (3.5)
	Urinary infection	4 (0.4)	0 (0.0)	4 (0.9)	7 (0.3)	3 (0.2)	4 (0.4)
	Sepsis	6 (0.6)	1 (0.2)	5 (1.1)	6 (0.3)	4 (0.3)	2 (0.2)
	Others	27 (2.7)	14 (2.6)	13 (2.8)	30 (1.3) *	14 (1.1)	16 (1.6)
	Total	240 (24.12)	163 (30.9)	76 (16.4)	172 (7.5) *	114 (9.0)	58 (5.7)
	Respiratory disease	7 (0.7)	3 (0.6)	4 (0.9)	15 (0.7)	10 (0.8)	5 (0.5)
	vascular disorder	78 (7.8)	40 (7.5)	38 (8.2)	100 (4.4) *	66 (5.2)	34 (3.3)
	Gastrointestinal	51 (5.1)	26 (4.9)	25 (5.4)	48 (2.1) *	27 (2.1)	21 (2.0)

	disorders						
Medical	Urological disorders	19 (1.9)	8 (1.5)	11 (2.4)	28 (1.2)	24 (1.9)	4 (0.4)
	Endocrine and metabolic diseases	53 (5.3)	33 (6.2)	20 (4.3)	49 (2.1) *	24 (1.9)	25 (2.4)
	tumors	39 (3.9)	24 (4.5)	15 (3.2)	82 (3.6)	52 (4.1)	30 (2.9)
	Others	10 (0.01)	6 (1.1)	4 (0.9)	33 (1.4)	13 (1.0)	20 (2.0)
	Total	257 (25.83)	140 (26.37)	117 (25.2)	355 (15.45) *	216 (16.9)	139 (13.6)
	Head trauma	9 (0.9)	5 (0.9)	4 (0.9)	5 (0.2) *	4 (0.3)	1 (0.1)
Trauma	Fracture	72 (7.2)	22 (4.1)	50 (10.8)	171 (7.4)	66 (5.2)	105 (10.2)
	Others	10 (1.0)	8 (1.5)	2 (0.4)	37 (1.6)	32 (2.5)	5 (0.5)
	Total	91 (9.15)	35 (6.59)	56 (12.1)	213 (9.27)	102 (8.0)	111 (10.8)
Total		995 (100)	531 (100)	464 (100)	2298 (100)	1273 (100)	1025 (100)

Primary diagnoses: I.e., the reason for hospitalization, which refers to the diagnosis with the greatest health hazard, most cost of treatment and longest hospitalization in this medical event; Male (%): the number of male patients with that primary diagnosis of a certain disease as a percentage of the number of all male patients; Female(%): the number of female patients with a primary diagnosis of a certain disease as a percentage of the number of all female patients; For re-admitted AD and PD patients,*$p<0.05$ indicated a statistically significant difference in gender distribution between AD and PD patients with the same primary diagnosis.

Table S4

Comparison of total first-admission costs (Yuan) between AD and PD.

Cost	first admission					
	AD			PD		
	Total	Male (SE)	Female (SE)	Total	Male (SE)	Female (SE)
care services	4509.94 (305.44)	5185.85 (488.32)	3736.43 (338.67)	2039.59 (125.55) *	2324.99 (210.61)	1685.14 (103.05)
medicine	8113.70 (801.25)	10150.92 (1427.97)	5782.31 (512.22)	3745.48 (183.73) *	4320.62 (283.68)	3031.19 (211.45)
examination/laboratory tests	12117.44 (612.43)	13382.51 (1012.79)	10669.7 (611.97)	7871.85 (188.66) *	8217.41 (287.19)	7442.68 (226.79)
surgery/inventory cost	4349.28 (523.13)	5137.33 (921.7)	3447.44 (380.26)	48770.77 (2097.75) *	49501.44 (2804.74)	47863.32 (3161.19)
total costs	29090.36 (1923.21)	33856.61 (3349.49)	23635.88 (1486.81)	62427.70 (2125.80) *	64364.46 (2852.55)	60022.33 (3187.93)

SE: Standard Error; Male (SE): means the total amount of money consumed by male patients in a certain service, expressed as an average (SE). Female (SE): means the total amount of money consumed by female patients in a certain service, expressed as an average (SE); For consumption in the same service area at first hospitalization, *$p < 0.05$ suggested statistically significant differences between the gender distribution of AD and PD patients.

Table S5

Age distribution of AD and PD on re-hospitalization.

	re-hospitalization					
Age	AD			PD		
	Total	Male (%)	Female (%)	Total	Male (%)	Female (%)
< 45	0 (0.0)	0 (0.0)	0 (0.0)	6 (1.6)	3 (1.4)	3 (1.8)
45-54	0 (0.0)	0 (0.0)	0 (0.0)	23 (6.2) *	11 (5.3)	12 (7.4)
55-64	2 (0.9)	1 (0.7)	1 (1.1)	67 (18.1) *	35 (16.8)	32 (19.6)
65-74	17 (7.4)	5 (3.6)	12 (13.0)	94 (25.3) *	48 (23.1)	46 (28.2)
75-84	56 (24.2)	22 (15.8)	34 (37.0)	127 (34.2) *	70 (33.7)	57 (35)
85+	156 (67.5)	111 (79.9)	45 (48.9)	54 (14.6) **	41 (19.7)	13 (8.0)
All ages	231 (100)	139 (100)	92 (100)	371 (100)	208 (100)	163 (100)

Male (%): The percentage of male patients in this age group in the total male population at the time of re-hospitalization; Female (%): The percentage of female patients in this age group in the total female population at the time of re-hospitalization; For re-admissions at the same age range, *$p < 0.05$ indicated statistical significance differences in gender distribution between AD and PD patients.

Table S6

Distribution of AD and PD patients in different hospital departments at re-admission

Admission wards	re-hospitalization					
	AD			PD		
	Total	Male (%)	Female (%)	Total	Male (%)	Female (%)
Neurology	11 (4.8)	9 (6.5)	2 (2.2)	141 (38.0) **	69 (33.2)	72 (44.2)
Geriatrics	173 (74.9)	115 (82.7)	58 (63.0)	70 (18.9) **	52 (25.0)	18 (11.0)
Psychiatric	13 (5.6)	1 (0.7)	12 (13.0)	20 (5.4)	8 (3.8)	12 (7.4)
Respiratory	8 (3.5)	5 (3.60)	4 (4.3)	9 (2.4)	5 (2.40)	4 (2.5)
Orthopaedics	7 (3.0)	2 (1.4)	5 (5.4)	22 (5.9)	13 (6.25)	9 (5.5)
Infectious	0 (0.0)	0 (0.0)	0 (0.0)	3 (0.8)	3 (1.4)	0 (0.0)
Rehabilitation	7 (3.0)	2 (1.4)	5 (5.4)	13 (3.5)	4 (1.9)	9 (5.5)
Urology	0 (0.0)	0 (0.0)	0 (0.0)	7 (1.9)	6 (2.9)	1 (0.6)
Medicine	11 (4.8)	6 (4.3)	5 (5.4)	62 (16.7) *	35 (16.8)	27 (16.6)
Surgery	0 (0.0)	0 (0.0)	0 (0.0)	9 (2.4)	7 (3.4)	2 (1.2)
Intensive care unit	0 (0.0)	1 (0.72)	0 (0.0)	2 (0.5)	1 (0.5)	1 (0.6)
Other	1 (0.4)	0 (0.0)	1 (1.1)	13 (3.5)	5 (2.4)	8 (4.9)
Total	231 (100)	139 (100)	92 (100)	371 (100)	208 (100)	163 (100)

Male (%): The percentage of male patients in this department in all male patients at the time of re-hospitalization; Female (%) The percentage of female patients in this department as a percentage of all female patients at the time of re-hospitalization; For re-admitted AD and PD patients, *$p < 0.05$ indicated a significant difference in

gender distribution between AD and PD patients hospitalized in the same department.

Table S7

Distribution of primary disease diagnoses at re-admission in AD and PD patients.

Reason	Specification	re-hospitalization					
		AD			PD		
		Total	Male (%)	Female (%)	Total	Male (%)	Female (%)
Neurological	AD or PD	34 (14.7)	14 (10.1)	20 (21.7)	134(36.1**	68 (32.7)	66 (40.5)
	Neuropsychiatric disorders	6 (2.6)	1 (0.7)	5 (5.4)	23 (6.2) *	9 (4.3)	14 (8.6)
	Neurogenetic degeneration/ Immune related disorders	6 (2.6)	3 (2.2)	3 (3.3)	6 (1.6)	1 (0.5)	5 (3.1)
	Cerebrovascular disease	16 (6.9)	11 (7.9)	5 (5.4)	11 (3.0)	8 (3.8)	3 (1.8)
	Others	1 (0.4)	0 (0.0)	1 (1.1)	5 (1.3)	2 (1.0)	3 (1.8)
	Total	63 (27.3)	29 (20.9)	34 (37.0)	179 (48.2) **	88 (42.3)	91 (55.8)
Infectious	Pulmonary infection	74 (32.0)	59 (42.4)	15 (16.3)	44 (11.9) **	34 (16.3)	10 (6.1)
	Urinary infection	5 (2.2)	2 (1.4)	3 (3.3)	2 (0.5)	2 (1.0)	0 (0.0)
	Sepsis	1 (0.4)	1 (0.7)	0 (0.0)	1 (0.3)	1 (0.5)	0 (0.0)
	Others	9 (3.9)	2 (1.4)	7 (7.6)	4 (1.1)	3 (1.4)	1 (0.6)
	Total	89 (38.5)	64 (46.0)	25 (27.2)	51 (13.7)	40 (19.2)	11 (6.7)
Medical	Respiratory disease	4 (1.7)	3 (2.2)	1 (1.1)	1 (0.3)	1 (0.5)	0 (0.0)
	vascular disorder	20 (8.7)	11 (7.9)	9 (9.8)	23 (6.2)	16 (7.7)	7 (4.3)
	Gastrointestinal disorders	8 (3.5)	4 (2.9)	4 (4.3)	8 (2.2)	4 (1.9)	4 (2.5)
	Urological disorders	6 (2.6)	4 (2.9)	2 (2.2)	6 (1.6)	6 (2.9)	0 (0.0)
	Endocrine and metabolic diseases	8 (3.5)	6 (4.3)	2 (2.2)	11 (3.0)	6 (2.9)	5 (3.1)
	tumors	12 (5.2)	10 (7.2)	2 (2.2)	29 (7.8)	20 (9.6)	9 (5.5)
	Others	1 (0.4)	0 (0.0)	1 (1.1)	12 (3.2)	5 (2.4)	7 (4.3)

	Total	59 (25.5)	38 (27.3)	21 (22.8)	90 (24.3)	58 (27.9)	32 (19.6)
Trauma	Head trauma	1 (0.4)	1 (0.7)	0 (0.0)	1 (0.3)	0 (0.0)	1 (0.6)
	Fracture	19 (8.2)	7 (5.0)	12 (13.0)	45 (12.1)	17 (8.2)	28 (17.2)
	Others	0 (0.0)	0 (0.0)	0 (0.0)	5 (1.3)	5 (2.4)	0 (0.0)
	Total	20 (8.7)	8 (5.8)	12 (13.0)	51 (13.7) *	22 (10.6)	29 (17.8)
Total		231 (100)	139 (100)	92 (100)	371 (100)	208 (100)	163 (100)

Primary diagnosis: i.e., reason for hospitalization, refers to the diagnosis with the greatest health hazard, highest cost of treatment and longest hospitalization during this medical event; Male (%): number of male patients with a primary diagnosis of a certain disease at re-admission as a percentage of all male patient numbers at re-admission; Female (%): the number of female patients with a primary diagnosis of a certain disease at re-admission as a percentage of all female patient numbers at re-admission; For re-admitted AD and PD patients, *$p < 0.05$ indicated a statistically significant difference in their gender distribution between AD and PD patients with the same primary diagnosis.

Table S8

Comparison of total re-hospitalization costs (Yuan) between AD and PD

Cost	re-hospitalization					
	AD			PD		
	Total	Male (SE)	Female (SE)	Total	Male (SE)	Female (SE)
care services	6231.44 (668.13)	5954.02 (627.75)	6650.59 (1388.09)	2970.63 (496.04) *	3826.18 (860.13) **	1870.06 (242.99) *
medicine	8735.06 (994.11)	10184.43 (1360.59)	6545.26 (1393.73)	4577.65 (591.59) *	5188.29 (848.7) *	3787.19 (794.06) *
examination/laboratory tests	12832.93 (854.09)	12872.75 (933.94)	12772.77 (1621.95)	8407.82 (502.91) *	8794.48 (777.28) *	7879.6 (569.38) *
surgery/inventory cost	3112.51 (461.66)	3040.38 (549.87)	3221.51 (812.16)	27040.02 (3999.48) *	18791.25 (4170.68) **	37368.89 (7277.11) *
total costs	30911.96 (2453.11)	32051.58 (2956.84)	29190.13 (4254.85)	43112.32 (4196.12) **	36777.02 (4658.46) *	50905.74 (7393.81) *

Yuan: the legal common currency of the people's Republic of China; SE: standard error; Male (SE): means the total amount of money consumed by male patients in a certain service. Female (SE): means the total amount of money consumed by female patients in a certain service; For consumption in the same service area of re-hospitalization, *$p < 0.05$ suggested statistically significant differences between the gender distribution of AD and PD patients.

Table S9

Analysis of the risk factors of AD re-hospitalization and intrahospital mortality

Disease	re-hospitalization						intrahospital mortality	
	univariate analysis		multivariable model				univariate analysis	
AD	F	P	Exp(B)	P	Adjusted OR (95% CI)		F	P
Sex	5.223	**0.023**					0.033	0.855
Age	80.012	**< 0.001**	0.932	**< 0.001**	0.911	0.953	1.262	0.262
hospitalization duration	34.906	**< 0.001**	0.988	**< 0.001**	0.981	0.995	0.202	0.654
comorbidity in hospital	0.005	0.946					0.095	0.758
neurological hospitalization	34.601	**< 0.001**	2.437	**< 0.001**	1.413	4.204	1.252	0.263
surgical procedures	15.616	**< 0.001**	0.523	**0.003**	0.342	0.800	0.070	0.791
re-hospitalization							0.001	0.970

Binary logistic regression model was used to analyze the risk factors associated with re-hospitalization and intrahospital mortality in AD patients. The presence or absence of re-admission and the presence or absence of death during hospitalization were used as covariates, and gender, age, length of stay, hospital comorbidity, department of hospitalization, and presence or absence of surgical procedures were used as covariates for statistical analysis. Age of AD patients, length of stay, choice of neurology hospitalization and surgical procedures were risk factors for re-admission of AD patients. in-hospital mortality of AD patients was not associated with all of the above covariates. Statistically significant results ($p < 0.05$) were highlighted in bold.

Table S10

Analysis of the risk factors of PD re-hospitalization and intrahospital mortality.

Disease	re-hospitalization						intrahospital mortality					
	univariate analysis		multivariable model				univariate analysis		multivariable model			
PD	F	P	Exp(B)	P	Adjusted OR (95% CI)		F	P	Exp(B)	P	Adjusted OR (95% CI)	
Sex	0.120	0.729					3.229	0.072				
Age	33.874	**< 0.001**	0.982	**0.001**	0.971	0.993	54.615	**< 0.001**	1.107	**< 0.001**	1.061	1.154
hospitalization duration	26.054	**< 0.001**	0.989	**0.012**	0.980	0.998	55.016	**< 0.001**	1.007	0.143	0.998	1.015
comorbidity in hospital	0.084	0.773					20.317	**< 0.001**	3.544	**0.019**	1.228	10.233
neurological hospitalization	28.629	**< 0.001**	1.505	**0.001**	1.1746	1.929	43.579	**< 0.001**	0.242	**0.005**	0.090	0.655
surgical procedures	12.321	**< 0.001**	0.661	**0.001**	0.521	0.838	0.078	0.780				
re-hospitalization							27.182	**< 0.001**	2.681	**0.003**	1.386	5.189

Binary logistic regression models were used to analyze the risk factors associated with re-admission and in-hospital mortality in -PD patients. The presence or absence of re-admission and the presence or absence of death during hospitalization were used as covariates, and gender, age, length of stay, hospital comorbidity, department of hospitalization, and presence or absence of surgical procedures were used as covariates for statistical analysis. When exploring factors influencing in-hospital mortality, we also included the presence or absence of re-admission as a covariate in the statistical analysis. Age, length of stay, neurological hospitalization, and surgical procedures were risk factors for re-admission in PD patients. The in-hospital mortality in PD patients was significantly associated with patients age, comorbidities, neurological hospitalization and a history of re-admission. Statistically significant results ($p < 0.05$) were highlighted in bold.
